# Supplementary material for: Modeling glioblastoma heterogeneity as a dynamic network of cell states
Source: Mol Syst Biol. 2021 Sep 16;17(9):e10105. doi: 10.15252/msb.202010105 (PMC8444284; doi:10.15252/msb.202010105)
Supplement: Supplementary file 5 — Source Data for Figure 3 [file MSB-17-e10105-s001.zip › Figure3A_sourcedata/GSEA_3065/hallmarks_state6.GseaPreranked.1623416308702/HALLMARK_PEROXISOME.html]

Details for gene set HALLMARK\_PEROXISOME[GSEA]

|  || Dataset | state6 |
| Phenotype | NoPhenotypeAvailable |
| Upregulated in class | na\_pos |
| GeneSet | HALLMARK\_PEROXISOME |
| Enrichment Score (ES) | 0.22930387 |
| Normalized Enrichment Score (NES) | 0.89037114 |
| Nominal p-value | 0.7036083 |
| FDR q-value | 0.7056253 |
| FWER p-Value | 1.0 |
Table: GSEA Results Summary

  

Fig 1: Enrichment plot: HALLMARK\_PEROXISOME      
 Profile of the Running ES Score & Positions of GeneSet Members on the Rank Ordered List

  

| PROBE | GENE SYMBOL | GENE\_TITLE | RANK IN GENE LIST | RANK METRIC SCORE | RUNNING ES | CORE ENRICHMENT || 1 | PABPC1 |  |  | 15 | 0.292 | 0.0733 | Yes |
| 2 | HRAS |  |  | 245 | 0.163 | 0.0889 | Yes |
| 3 | YWHAH |  |  | 449 | 0.128 | 0.0986 | Yes |
| 4 | TSPO |  |  | 474 | 0.126 | 0.1281 | Yes |
| 5 | FIS1 |  |  | 530 | 0.120 | 0.1527 | Yes |
| 6 | BCL10 |  |  | 588 | 0.115 | 0.1757 | Yes |
| 7 | PRDX5 |  |  | 729 | 0.103 | 0.1861 | Yes |
| 8 | CNBP |  |  | 997 | 0.086 | 0.1776 | Yes |
| 9 | SCP2 |  |  | 1067 | 0.082 | 0.1907 | Yes |
| 10 | ITGB1BP1 |  |  | 1119 | 0.080 | 0.2053 | Yes |
| 11 | SOD2 |  |  | 1228 | 0.075 | 0.2121 | Yes |
| 12 | CTBP1 |  |  | 1244 | 0.074 | 0.2293 | Yes |
| 13 | ECI2 |  |  | 1525 | 0.063 | 0.2134 | No |
| 14 | ALDH9A1 |  |  | 1537 | 0.063 | 0.2282 | No |
| 15 | ISOC1 |  |  | 2024 | 0.047 | 0.1846 | No |
| 16 | ACAA1 |  |  | 2308 | 0.038 | 0.1620 | No |
| 17 | ABCC5 |  |  | 2430 | 0.035 | 0.1572 | No |
| 18 | IDH1 |  |  | 2508 | 0.033 | 0.1569 | No |
| 19 | SIAH1 |  |  | 2529 | 0.033 | 0.1629 | No |
| 20 | VPS4B |  |  | 2884 | 0.024 | 0.1287 | No |
| 21 | ACSL1 |  |  | 3113 | 0.020 | 0.1076 | No |
| 22 | ERCC3 |  |  | 3166 | 0.018 | 0.1064 | No |
| 23 | ECH1 |  |  | 3187 | 0.018 | 0.1087 | No |
| 24 | ACSL4 |  |  | 3290 | 0.016 | 0.1010 | No |
| 25 | SMARCC1 |  |  | 3546 | 0.010 | 0.0745 | No |
| 26 | SLC23A2 |  |  | 3562 | 0.010 | 0.0753 | No |
| 27 | PEX2 |  |  | 3615 | 0.009 | 0.0717 | No |
| 28 | ACOT8 |  |  | 3632 | 0.008 | 0.0720 | No |
| 29 | ERCC1 |  |  | 3807 | 0.005 | 0.0532 | No |
| 30 | CLN8 |  |  | 3840 | 0.004 | 0.0506 | No |
| 31 | CAT |  |  | 3983 | 0.001 | 0.0346 | No |
| 32 | ABCD3 |  |  | 4080 | -0.001 | 0.0238 | No |
| 33 | CDK7 |  |  | 4198 | -0.003 | 0.0112 | No |
| 34 | MSH2 |  |  | 4317 | -0.005 | -0.0010 | No |
| 35 | IDE |  |  | 4407 | -0.007 | -0.0094 | No |
| 36 | GNPAT |  |  | 4548 | -0.010 | -0.0229 | No |
| 37 | PEX13 |  |  | 4933 | -0.017 | -0.0625 | No |
| 38 | HSD17B4 |  |  | 4950 | -0.017 | -0.0599 | No |
| 39 | CRAT |  |  | 5200 | -0.022 | -0.0827 | No |
| 40 | RDH11 |  |  | 5274 | -0.024 | -0.0850 | No |
| 41 | PRDX1 |  |  | 5508 | -0.029 | -0.1044 | No |
| 42 | ACOX1 |  |  | 5517 | -0.029 | -0.0979 | No |
| 43 | CTPS1 |  |  | 5522 | -0.029 | -0.0909 | No |
| 44 | MVP |  |  | 5641 | -0.031 | -0.0964 | No |
| 45 | HMGCL |  |  | 5765 | -0.034 | -0.1018 | No |
| 46 | SLC25A17 |  |  | 6109 | -0.040 | -0.1307 | No |
| 47 | GSTK1 |  |  | 6180 | -0.042 | -0.1279 | No |
| 48 | PEX6 |  |  | 6622 | -0.053 | -0.1647 | No |
| 49 | CLN6 |  |  | 6939 | -0.063 | -0.1847 | No |
| 50 | SLC35B2 |  |  | 6978 | -0.064 | -0.1726 | No |
| 51 | IDH2 |  |  | 6985 | -0.064 | -0.1568 | No |
| 52 | SOD1 |  |  | 7066 | -0.067 | -0.1489 | No |
| 53 | ELOVL5 |  |  | 7151 | -0.069 | -0.1407 | No |
| 54 | HSD17B11 |  |  | 7363 | -0.077 | -0.1452 | No |
| 55 | MLYCD |  |  | 7368 | -0.077 | -0.1260 | No |
| 56 | TOP2A |  |  | 7430 | -0.079 | -0.1128 | No |
| 57 | SLC25A4 |  |  | 7519 | -0.082 | -0.1017 | No |
| 58 | FDPS |  |  | 7587 | -0.085 | -0.0875 | No |
| 59 | IDI1 |  |  | 7851 | -0.099 | -0.0924 | No |
| 60 | LONP2 |  |  | 8041 | -0.110 | -0.0859 | No |
| 61 | FADS1 |  |  | 8186 | -0.120 | -0.0714 | No |
| 62 | CADM1 |  |  | 8282 | -0.130 | -0.0490 | No |
| 63 | SEMA3C |  |  | 8293 | -0.131 | -0.0164 | No |
| 64 | DHCR24 |  |  | 8321 | -0.134 | 0.0150 | No |
| 65 | ATXN1 |  |  | 8456 | -0.153 | 0.0389 | No |
Table: GSEA details [plain text format]

  

Fig 2: HALLMARK\_PEROXISOME: Random ES distribution      
 Gene set null distribution of ES for **HALLMARK\_PEROXISOME**
